# Immunological and Pathological Landscape of Dengue Serotypes 1-4 Infections in Immune-Competent Mice

**DOI:** 10.3389/fimmu.2021.681950

**Published:** 2021-06-08

**Authors:** Abhay P. S. Rathore, Chinmay K. Mantri, Meredith W. Tan, Roksana Shirazi, Andrew Nishida, Siti A. B. Aman, Juliet Morrison, Ashley L. St. John

**Affiliations:** ^1^ Program in Emerging Infectious Diseases, Duke-National University of Singapore, Singapore, Singapore; ^2^ Department of Microbiology and Plant Pathology, University of California, Riverside, CA, United States; ^3^ Department of Microbiology, University of Washington, Seattle, WA, United States; ^4^ Department of Pathology, Duke University Medical Center, Durham, NC, United States; ^5^ Department of Microbiology and Immunology, Young Loo Lin School of Medicine, National University of Singapore, Singapore, Singapore

**Keywords:** dengue, DENV 1-4, transcriptomics, spleen, liver, flow cytometry, histology, fibroblast reticular cell

## Abstract

Dengue virus (DENV), a Flavivirus, causes a broad spectrum of disease in humans with key clinical signs including thrombocytopenia, vascular leakage and hemorrhaging. A major obstacle to understanding DENV immunity has been the lack of a validated immune-competent mouse model. Here, we report the infection profiles of human clinical isolates of DENV serotypes 1-4 in an immune-competent mouse model. We detected replicating DENV in the peritoneal cells, liver and the spleen that was generally resolved within 2 weeks. The DENV target cell types for infection were monocytes/macrophages, dendritic cells, endothelial cells, and we identified a novel DENV cellular target, fibroblast reticular cells of the spleen. We observed gross pathologies in the spleen and liver that are consistent with dengue disease, including hemorrhaging as well as transcriptional patterns suggesting that antiviral responses and tissue damage were induced. Key clinical blood parameters that define human DENV disease such as hemoconcentration, leukopenia and reduced number of platelets were also observed. Thus, immune-competent mice sustain replicating infection and experience signs, such as hemorrhaging, that define DENV disease in humans. This study thoroughly characterizes DENV1-4 infection in immune-competent mice and confirms the wild-type mouse model as a valid and reproducible system for investigating the mechanisms of DENV pathogenesis.

## Introduction

Dengue virus (DENV) is a Flavivirus that causes substantial worldwide morbidity and mortality (1). This virus is spread by *Aedes* mosquito vectors and, with natural infections, causes clinically apparent disease only in humans. Recent reports have shown that the worldwide burden of dengue is much greater than previously thought, with approximately 390 million cases per year (2). This under-diagnosis is potentially due to several factors, such as the possibility that some individuals with clinically apparent but mild disease are not recognized as having dengue, or that more individuals are asymptomatic during dengue infection than was initially understood (2). While this observation indicates that the global burden of dengue may be higher than prior estimates showed, it also raises the possibility that the spectrum of clinical disease is wider than was earlier thought, with more cases presenting as asymptomatic or sub-clinical. Signs and symptoms of dengue fever (DF), the mild form of disease, include an acute febrile response, rash, and thrombocytopenia (3). Even patients with DF can experience mild bleeding complications, such as an increased likelihood of bruising (4). Patients with severe dengue, also known as dengue hemorrhagic fever and dengue shock syndrome (DHF/DSS) may have overt hemorrhaging that can lead to shock and death. During infection, DHF/DSS patients may experience accumulation of fluid within body cavities and damage to organs as a result of edema. If untreated, dengue hemorrhaging can cause death, either due to organ damage or hypovolemic shock (3).

To date, one of the largest obstacles for understanding the mechanisms of dengue immunity and pathogenesis has been the lack of a good animal model (5). Much of our understanding of how the virus spreads *in vivo* has been revealed experimentally in primates, including monkeys and humans. For example, the sequential targeting of lymph nodes by DENV to reach systemic disease was shown in monkeys (6). Key cellular targets of dengue infection, such as dendritic cells (DCs), Langerhans cells, monocytes, macrophages and, controversially, endothelial cells were either experimentally defined using human cells or identified by histological analysis using tissues from dengue patients, frequently upon autopsy (7, 8). To understand the correlates of protection and mechanisms of disease, an animal model that can be experimentally manipulated has been an aim of the dengue field since its inception, with the mouse model being most desired (5).

Historically, it has been stated that DENV does not cause infection of mice that are immune-competent, although there is a growing body of literature that supports DENV infection in immune-competent mice (9–13). Many of those studies claiming that immune-competent mice do not support replicating infection were also performed in the earlier days of the dengue field, prior to the development of sensitive molecular techniques to assess virus replication *in vivo* and outcomes such as vascular leakage or cellular immune responses (5, 14). To circumvent the obstacle posed by the lack of a good mouse model, immune-compromised mice (especially the AG129 model that lacks receptors for IFN-α\β & IFN-γ) have been used in recent years to study dengue (15). These models have revealed important information regarding DENV pathogenesis. For instance, it was experimentally demonstrated using the AG129 model that antibody-dependent enhanced replication of DENV can occur *in vivo*, both due to pre-existing antibodies and due to maternal transfer of antibodies (16, 17). These experiments proved that antibody-dependent enhancement of replication, the primary theory for increased likelihood of severe disease during secondary DENV infection (at least in certain human populations), is possible and can occur *in vivo*. Yet, immune-compromised mice also have important limitations. For example, that they experience severe pathology and uniformly lethal infection to certain challenge strains is not consistent with our current epidemiological understanding of the clinical spectrum of dengue disease experienced by humans, where the vast majority of patients also have mild or asymptomatic dengue (18, 19). More recently, mice that lack IFN in specific cellular compartments (20) have advanced this model, but limitations still remain. Particularly with respect to studying immune correlates of protection and immune-mediated pathology, the immune-compromised systems have caveats for interpretation since it is not known if the same mechanisms of disease would occur in immune-competent hosts. Furthermore, the IFN system is important for processes such as B cell activation and antibody production (21–23), which could influence protective immune responses. Ideally, an animal model for dengue should sustain virus replication and also recapitulate the clinical features of dengue disease, including vascular pathology, which is a distinguishing presentation of dengue that is thought to result from immune pathology.

Increasingly, there are reports that dengue can infect wild-type mice (9, 11, 24, 25) but this observation has remained controversial. Here, we aimed to characterize and compare the growth kinetics of representative strains of each of the 4 serotypes of DENV *in vivo* in the mouse model and to define the hematological parameters of infection, the target organs and infected cell types. In the process, we observed a disease course reminiscent to human disease occurring in wild-type mice, since all four serotypes replicated *in vivo* and induced key signs of disease such as thrombocytopenia, leukopenia, vascular leakage, and tissue damage, including in the liver. The cell types infected in target organs were also similar to those that have been identified as infected in humans, including DCs, monocytes/macrophages, and endothelial cells. Furthermore, our detailed study has revealed reticular fibroblast cells of the spleen as a novel cellular target in the mouse model of dengue disease. Transcriptomic analysis from liver and spleen further validated DENV infection and highlighted pathways enriched for tissue damage and infection control.

## Materials and Methods

### Dengue Virus Propagation and Quantification

DENV serotypes 1-4 are clinical isolates, originally obtained from the EDEN study (26) (DENV1 accession no. EU081230.1: DENV2 accession no: EU081177.1, DENV3 accession no.: EU081190.1, DENV4 accession no.: GQ398256.1). Viruses were amplified in the *Aedes albopictus* C6/36 mosquito cell line (ATCC). Briefly, ~80% confluent, C6/36 cells were infected with DENV1-4 and incubated at 28°C in the maintenance media for 5 days. To harvest the virus, supernatants were clarified using centrifugation, portioned in small volume aliquots and stored at -80°C. For concentration of virus stocks to achieve high titer for subcutaneous injection, virus was ultracentrifuged at 20,000 RPM for 2 hours at 4°C. Pellet was then resuspended with RPMI and cushioned on 30% sucrose and ultracentrifuged at 25,000 RPM for 4 hours on a Beckman SW28 swinging bucket. Virus pellet was rehydrated overnight at 4°C in 1ml PBS and stored in small aliquots at -80°C. All viruses used in this study were low passage (<10) clinical isolates.

For virus quantifications, plaque assay was carried out to determine the virus titer for each serotype. Baby hamster kidney (BHK-21, from ATCC) cells were seeded at a density of (2x10^5^) per well in a 24-well plate. The next day, virus was serially diluted in serum-free media and added to each well and incubated at 37°C in 5% CO_2_ for 2h. At 2h, virus-containing media was then aspirated and supplemented with 0.5mL/well of carboxy-methyl-cellulose (CMC) overlay media, followed by incubation for 5 days. Following which, the plate was submerged in paraformaldehyde (PFA) for two hours to fix cells then rinsed before adding 300μL of crystal violet to each well. The virus titer was calculated by counting plaques and expressed in plaque-forming units, PFU/ml.

### Infection of Mice

Female specific-pathogen-free C57/BL6 WT mice (InVivos, Singapore) or AG129 mice (a gift from the colony of Sylvie Alonso, maintained by InVivos, Singapore) 6-10 weeks of age were injected intraperitoneally (I.P.) with 100μL of approximately 1x10^6^ PFU of DENV-1 to -4 and the time course of infection was followed until day 14 post-infection. Alternatively, subcutaneous injection was performed in rear footpads using 8x10^8^ PFU of DENV2 in a 20uL volume of saline. At various time points post-infection blood was collected for whole blood analysis, serum was isolated for NS1 quantification and organs were harvested to measure viral load.

### Immunostaining of Tissue Sections

Spleens were frozen in OCT compound (Tissue-Tek) prior to sectioning to 10μm thickness using a cryostat (Leica). Sections were acetone fixed at 4°C for 20 min, allowed to dry, then re-hydrated with PBS containing 1% BSA. Sections were stained with primary antibodies, including ER-TR7 (Abcam) for FRCs and with an antibody against DENV NS3 (GeneTex). After washing with PBS, secondary antibodies including anti-rabbit AF546 and anti-rat AF488 (Thermo Scientific), were incubated for 2h at room temperature prior to washing. Slides were mounted with Prolong gold antifade reagent (Thermo Fisher Scientific). Images were acquired using a Carl Zeiss LSM710 confocal microscope. ImageJ software was used to generate the colocalization images.

### Whole Blood Analysis and Liver Function Tests

For whole blood analysis, mice were bled from the facial vein using a lancet. Blood was collected in EDTA tubes to prevent clotting (BD Biosciences), briefly vortexed and whole blood analysis was carried out using an A^C^T diff analyzer (Beckman Coulter) immediately after blood collection. For each blood sample, two analytical readings were taken and averaged. ALT Activity Assay kit (MAK052, Sigma-Aldrich) and AST Activity Assay kit (MAK055, Sigma-Aldrich) were used to perform liver function tests.

### DENV Genome Quantification in Tissues

At each time point, mice were sacrificed *via* CO_2_ euthanasia and the peritoneal lavage fluid, liver and spleen were immediately harvested. Peritoneal cells were pelleted by centrifugation at 1,000 x *g* for 5 min and resuspended in 1mL of PBS before counting, using a hemocytometer. Total RNA from the cells was isolated using RNeasy kit (Qiagen) following manufacturer instructions. Similarly, total RNA was also extracted from tissues using RNeasy kit (Qiagen). Briefly, the left lobe of the liver and whole spleen were collected from each mouse and weighed. Approximately 30mg of tissue was homogenized with RLT buffer and ceramic beads using the TissueLyser-II (Qiagen) at a frequency of 25.0 Hz for four minutes, incubating the tubes on ice at one-minute intervals to prevent RNA degradation. Following tissue homogenization, tubes were centrifuged at the maximum speed for 3 min and the supernatants were transferred to a clean eppendorf tube to be used for RNA extraction. Column-bound RNA was eluted in 40μL of nuclease-free water and quantified using a Nanodrop (Thermoscientific). RNA was isolated from control, uninfected tissues to verify the specificity of detection.

The cDNA was synthesized from 1μg of total RNA template using the iScript cDNA Synthesis Kit (Biorad), using sequence specific primers listed in **Supplementary Table 1**. Real-time PCR was performed using SYBR Green reagent (Biorad) in a 10μL reaction volume containing 100ng of cDNA template, and 0.1μL of 10μM forward and reverse primers that were optimized for each serotype and tissue. The thermocycler reaction conditions used were 3 min at 95°C, 40 cycles of (95°C for 10 sec, 55°C for 30 sec, 72°C for 15 sec), 10 sec at 95°C, followed by a melting curve from 55°C to 95°C at five second intervals of 0.5°C increment. For detection using probe SsoAdvanced™ Universal Probes Supermix (Biorad) was used in a similar reaction mixture containing 0.1μL of 10 μM probe. The cyclic conditions used were 3 min at 95°C, 40 cycles of (95°C for 10 sec, 62°C for 45 sec). For detection of –ve strand cDNA was of synthesized using iScript Select cDNA synthesis kit using a sense primer 5’-AAT ATG CTG AAA CGC GAG AGA AAC CGC G-3’ followed by PCR with primer pair 5’-AAT ATG CTG AAA CGC GAG AGA AAC CGC G-3’ and 5’-CCC ATC TCI TCA IIA TCC CTG TTG G-3’ to amplify a 170bp region from Capsid-PrM region of DENV genome.

Finally, virus titers were quantified by the standard curve method using plasmids containing the DENV-1 to -4 genome sequences. qPCR products were run on 2% agarose gels to visualize the bands of expected size and also to verify that no background detection bands occurred after amplification using control (uninfected) tissues. Moreover, expected size amplicons were also confirmed by using nucleotide sequencing. Experiments were performed a minimum of 2 repeats with n≥5 animals per experiment.

### RNA Isolation and Microarray Processing

RNA was extracted from livers and spleens of DENV- and mock-infected C57B/6NTac mice in triplicate. The Mouse Whole Genome Microarray 4x44K kit (Agilent Technologies) was used for probe labeling and microarray slide hybridization of each biological replicate. Slides were scanned on an Agilent DNA microarray scanner (model G2505B) using the XDR setting, and raw images were analyzed by using Agilent Feature Extraction software (version 9.5.3.1). Extracted liver and spleen sample raw data were partitioned and background corrected separately by using the “norm-exp” method with an offset of 1, and quantile normalized using the limma package in R. Probes were filtered for low intensity, requiring at least two samples with intensity above a threshold set at the 5% quantile for intensity. Probes were mean summarized by gene.

### Identification of Differentially-Expressed Genes and Functional Enrichment

DENV-infected replicates were compared to mock-infected samples based on a linear model for each gene using limma. The differential expression criteria were an absolute log2 fold change (LFC) of 0.58 and an adjusted P value of 0.05, calculated using a moderated t test with subsequent Benjamini-Hochberg correction. Ingenuity Pathway Analysis Knowledge Base (IPA; Ingenuity Systems) was used to functionally analyze statistically significant gene expression changes. All enrichment scores were calculated by IPA, using the probes that passed our quality control (QC) filter as the background data set.

### Identification of Infected Cells by Flow Cytometry

To quantify various populations of DENV positive cells in the spleen, spleen tissue was processed to a single cell suspension using collagenase treatment. Surface staining of DCs, macrophages, endothelial cells and reticular fibroblast cells was done using Pacific blue-conjugated anti-cd11c, PE-conjugated anti-cd11b, APC-conjugated anti-cd31, and the primary antibody ERTR7. The secondary antibody anti-rat AF488 (Thermo Scientific) was used to detect ERTR7. Cells were fixed using 4% paraformaldehyde prior to intracellular staining. For intracellular staining of DENV infected cells, cells were permeabilized using 0.1% saponin and primary antibody against DENV non-structural protein 3 (NS3, Genetex) was used. Anti-rabbit AF594 was used as a secondary stain to detect NS3. Flow cytometry was performed using multicolor BD LSRFortessa Analyzer (BD Biosciences) and data was analyzed using FlowJo software.

### Histology

For histology, mouse tissues (liver and spleen) were embedded in OCT and stored at -80°C. Tissue cross-sections of 15 μm thicknesses were cut using a cryostat and air-dried. Sections were stained with Hematoxylin and Eosin stain to visualize cellular infiltrations, edema and hemorrhaging in the tissues. All images are representative of observations from 3 or more independent experiments.

### NS1 Quantification

Quantification of soluble NS1 in the mouse serum was performed using Platelia DENV NS1 ELISA kit (Biorad) by following the manufacturer’s instructions. For quantitative analysis, purified NS1 from each of the serotypes 1-4 was used to establish standard curves for accurate serotype specific quantification.

## Results

### DENV Serotypes 1-4 Cause Systemic Infection in Immune-Competent Wild Type Mice

**Figure 1:**
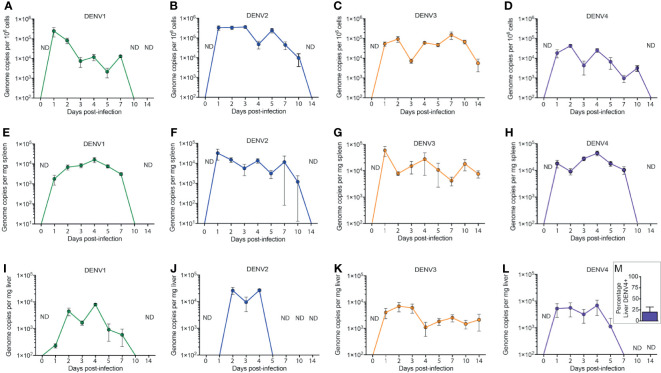
Systemic replication of DENV1-4 in WT mice. Mice (minimum of n=5 for each time point) were injected with 1x10^6^ pfu of DENV1-4 *via* intraperitoneal route and at various time points post-infection (days 0 to 14), cells of the peritoneum, spleen and liver were harvested for viral quantification. Quantification of viral genome copies demonstrates that viremia persisted for up to 10 days **(A–D)** in the cells of the peritoneum and for 5-7 days in the tissues such as the spleen **(E–H)** and the liver **(I–M)**. For **(M)**, the average percentage of DENV+ liver among DENV4-infected animals for three independent experiments, each with n=5 animals per experiment, is shown. Baseline values are shown as day 0. ND, not detected. The threshold of detection for all strains was approximately 10^1^, below the y-axis on the graphs.

To establish DENV infection in WT mice, animals were inoculated with 1x10^6^ plaque forming units (PFU) of DENV 1-4. At various time points post-infection, known DENV target organs such as the spleen and liver were harvested for quantification of virus replication. Since virus inoculation was delivered through the intra-peritoneal route to achieve systemic infection, cells from the peritoneum were also harvested for virus quantification. Quantification of viruses in the tissues was performed using real time RT-PCR due to its high sensitivity as a detection method. We found that due to the complexities in DENV genome as well as in the mouse genome, a single set of primers could not specifically detect all DENV serotypes in WT mouse tissues. Therefore, in this study, we have extensively optimized the primer sets that could detect the DENV genome with minimal background in specific tissues for each DENV serotype. This full list of primers for tissue and serotype specificities are presented in **Supplementary Table 1**. No virus was detected in uninfected tissues (time=0 in **Figure 1**, **Supplementary Figure 1**). DENV replication in cells of the peritoneal cavity after infection was bi-phasic for serotypes DENV1-4 with the first peak representing an immediate replication that occurred in peritoneal cells that was highest for DENV1 and DENV2 (**Figures 1A–D**) compared to DENV3 and DENV4 (**Figures 1C, D**). DENV2 infection in the peritoneum reached approximately 4x10^5^ genome copies/million cells at day 3 (**Figure 1B**). The second peak of replication for DENV1-4 occurred within the range of days 4-7 before virus was completely cleared at the initial site of infection, suggesting a second phase of infection from the progeny of early replication (**Figures 1A–D**).

DENV infection in the spleen was detected at an early time point, 24h post-infection for all four DENV serotypes, with peak titers achieved between days 1 and 4 post-infection (**Figures 1E–H**). The viral genome copy numbers in the spleens of DENV2, 3 or 4 infected mice (**Figures 1F–H**) were higher compared to those observed for DENV1 infection (**Figure 1E**). Similar to the peritoneum, the DENV1-4 replication profile in the liver was also bi-phasic with virus replication peaking at days 2 and 4 post-infection (**Figures 1I–L**). DENV3 and DENV4 (**Figures 1K, L**) both had very low levels of detectable viruses in the liver compared to DENV1 and DENV2 (**Figures 1I, J**), which had a higher liver burden. DENV2 replication was highest in the liver compared to other DENV serotypes (**Figure 1J**). Yet, despite a lower titer, DENV3 infection persisted longer than the other serotypes (**Figure 1K**). Furthermore, unlike DENV1-3, which were consistently detected in the liver, DENV4 was only detected in a minority of animals infected (**Figure 1M**). Interestingly, the kinetics of DENV3 infection in mice were extended, lasting beyond day 10 in the spleen and liver and still detectible in some mice at day 14 post-infection, compared to that of DENV1, 2 and 4 where virus was completely cleared in both of these secondary organs by day 10 (**Figure 1**). Since immune-compromised mice are more routinely used to study DENV, we performed a side-by-side comparison of DENV2 quantification in C57BL/6 versus AG129 mice (**Supplementary Figure 2**). As expected, the DENV burden was significantly higher in the peritoneal cavity, the site of injection, as well as in the serum, spleen and liver by 48h post-infection (**Supplementary Figure 2**). In contrast, UV-inactivated virus could not be detected systemically after inoculation (**Supplementary Figure 2**), supporting that there are significant increases in DENV genome copies detected over input levels. We also confirmed that infection could be initiated in WT mice by sub-cutaneous injection of virus, to approximate the natural route infection, which occurs when virus is deposited in the skin during a mosquito bite. Although a higher inoculating titer of 8x10^8^ PFU was used for DENV2, virus could be detected in the skin, draining lymph node, spleen, liver and serum for the 3 days of after infection which were monitored (**Supplementary Figure 3**). These results confirm WT mice can be productively infected *via* multiple routes and provide context for how the infection burden compares to immune-compromised mice.

**Figure 2:**
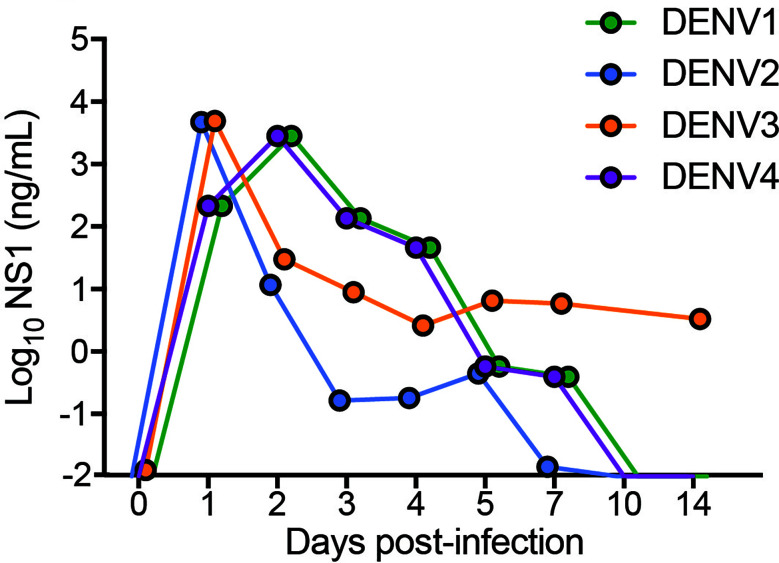
Detection and quantification of secretory non-structural protein, NS1 in the serum of DENV1-4 infected WT mice. Sera from mice infected with each of the DENV serotypes, DENV1-4 was collected at indicated time points for 14 days and soluble NS1 was quantified in the serum at each time points post-infection. NS1 antigen was present in all the groups (DENV1-4) for a period of 5-7 days peaking at days 2-3 for DENV1 and DENV4 and at day 1 for DENV2 and DENV3 respectively. Baseline values are shown as day 0.

To validate by another method that active replication of DENV occurs *in vivo* in mice, we quantitated the amounts of soluble non-structural protein 1 (sNS1) in the serum of mice each day of infection, since this protein is produced and secreted during replicating infection (27, 28). For all strains, sNS1 was detected and peaked within 24-48h post-infection (**Figure 2**). For DENV3, consistent with virus titers in the tissue (**Figure 1**), sNS1 was detected for a prolonged time course compared to the other serotypes, although at low levels (**Figure 2**). We also validated that replication of the virus genome occurs by detecting the negative-strand of the DENV2 genome in the peritoneal cells, PBMCs isolated from blood, spleen and liver (**Supplementary Figure 4**). Together, our data show that WT mice are susceptible to DENV1-4 infection and support active virus replication in key DENV target tissues. However, we also observed variability in the infection profile and kinetics for the unique strains of each serotype that were used.

### Transcriptional Reprogramming Occurs in DENV Target Organs

**Figure 3:**
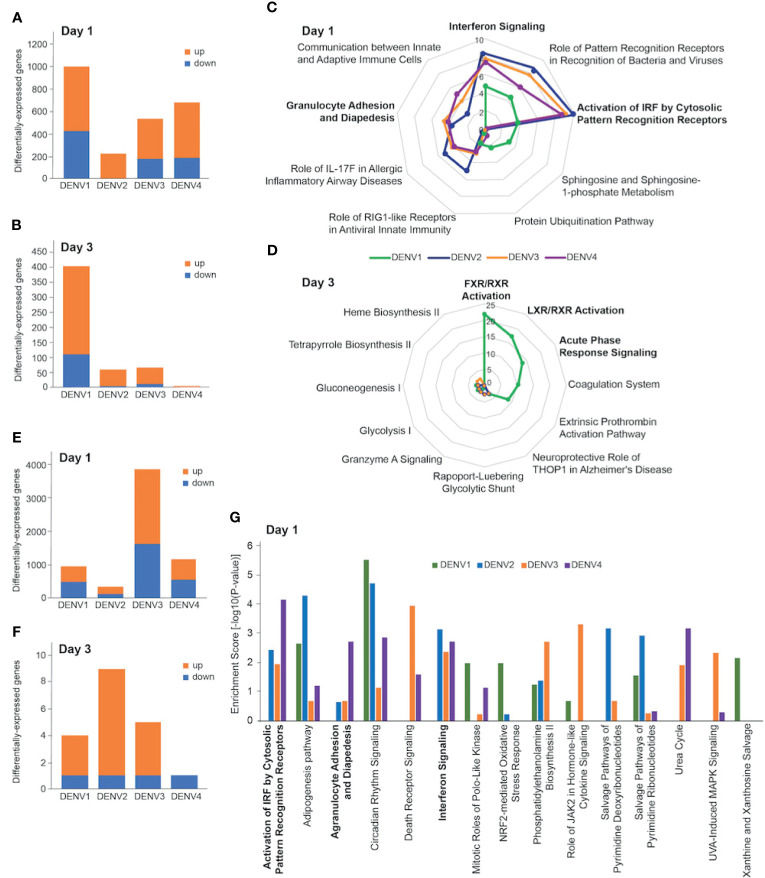
Transcriptional profiling of DENV-infected liver and spleen. Differentially-expressed genes (DEGs) in spleen **(A, B)** and liver **(E, F)** at day 1 **(A, E)** and day 3 **(B, F)** post infection with DENV1-4. Criteria used for differential expression analysis were an adjusted P value of <0.05, as determined by the limma empirical Bayes-moderated t test, and a log2FC of >0.58. DEGs were analyzed by IPA to produce lists of host pathways that were perturbed by the 4 serotypes. The top 5 enriched IPA canonical pathways in the spleen per DENV serotype on day 1 **(C)** and day 3 **(D)** are represented as radial plots. The distance from the center in each radial plot represents the enrichment score, which is defined as -log10(p value), using a right-tailed Fisher exact test. The top 5 enriched Ingenuity canonical pathways in the liver per DENV serotype on day 1 **(G)**.

To identify host drivers of this variability amongst the infection outcomes, we compared spleen transcriptomes of DENV-infected mice at days 1 and 3 post infection. As we had previously shown with DENV2 [13], most of the gene expression changes occurred on day 1 post infection (**Figure 3A**). The numbers of differentially-expressed genes (DEGs) varied depending on the DENV serotype and the organ profiled. There were fewer DEGs in the spleen (**Figures 3A, B**) compared with the liver across the 4 serotypes (**Figures 3E, F**). The pattern of gene expression also differed, with DENV1 infection causing the most robust response on day 1 with 576 upregulated and 418 downregulated genes (**Figure 3A**). This was followed by DENV4 (498 upregulated, 181 downregulated), and then DENV3 (362 upregulated, 174 downregulated). DENV2 induced the smallest gene expression change on day 1 with 213 upregulated genes and 1 downregulated gene (**Figure 3A**). On day 3 post infection, DENV1 induced the most gene expression changes (295 upregulated, 110 downregulated) followed by DENV3 (56 upregulated, 12 downregulated), DENV 2 (55 upregulated, 6 downregulated) and DENV4 (3 upregulated, 0 downregulated) (**Figure 3B**). We noted that the serotype that induced the most DEGs in the spleen, DENV1, was also the serotype that had the lowest peak infection in that organ (**Figure 1**).

When we subjected the splenic DEGs to pathway analysis, we found that the enrichment scores for the day 1 canonical pathways distinguished DENV1 from the other serotypes. DENV2-4 had high enrichment scores for innate immune pathways such as “Interferon Signaling”, “Granulocyte Adhesion and Diapedesis”, and “Activation of IRF by Cytosolic Pattern Recognition Receptors” (**Figure 3C**). The top canonical pathways that were unique to DENV1 on day 1 were “Sphingosine and Sphingosine-1-phosphate Metabolism”, and “Protein Ubiquitination Pathway” (**Figure 3C**). Only DENV1 induced large enough gene expression changes on day 3 for robust pathway analysis, which was just prior to the peak infection burden on day 4. The canonical pathways that were enriched included “FXR/RXR Activation”, “LXR/RXR Activation”, and “Acute Phase Response Signaling” (**Figure 3D**).

We next compared the liver transcriptional changes in DENV-infected mice. DENV3 induced the most robust response on day 1 with 2233 upregulated and 1618 downregulated genes (**Figure 3E**). This was followed by DENV4 (638 upregulated, 532 downregulated) and DENV1 (445 upregulated, 486 downregulated), which induced less than one third the number of DEGs that DENV3 did (**Figure 3E**). DENV2, the serotype that gave the highest viral burden in the liver (**Figure 1J**), induced the smallest gene expression change in the liver with 192 and 128 upregulated and downregulated genes respectively (**Figure 3E**). On day 3 post infection, few genes were differentially expressed in the livers of mice infected with any of the serotypes. DENV1-4 each downregulated 1 gene, while DENV1, DENV2, DENV3 and DENV4 upregulated 3, 8, 4 and 0 genes, respectively (**Figure 3F**).

Liver transcriptional responses differed depending on the DENV serotype. In DENV1 infection, there was no enrichment in innate immune pathways such as “Activation of IRF by Cytosolic Pattern Recognition Receptors”, “Interferon Signaling”, and “Agranulocyte Adhesion and Diapedesis”, (**Figure 3G**). However, DENV2-4 infection perturbed these pathways (**Figure 3G**). These results are generally consistent with the lower replication and clearance of DENV1 in the liver (**Figure 1I**), as lower virus loads lead to less virus recognition and the resulting interferon response. In contrast, higher viral burden in DENV2 liver was associated with an enrichment of pathway consistent with tissue necrosis (**Supplementary Figure 5**). Since DENV3 infection was prolonged in both spleen and liver (**Figure 1**), we questioned if unique transcriptomic signatures could characterize this persistence and identified genes and pathways associated with immune cell migration into the tissues (**Supplementary Figure 6**).

### DENV Infection of Mice Induces Dengue-Characteristic Hematological Changes

**Figure 4:**
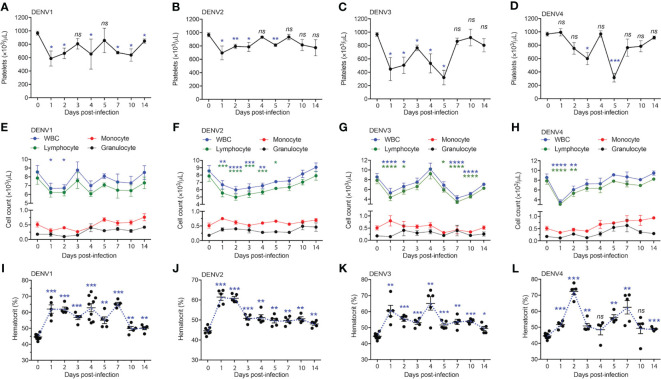
Full blood count shows thrombocytopenia, leukopenia and increase in the hematocrit of mice infected with serotypes, DENV1-4. Blood from mice (n=5-10) infected with DENV1-4 was collected at various days post-infection and subjected to complete blood count measurements. **(A–D)**, shows significant drop in the platelet counts starting from days 1-5 for each of the DENV serotypes. **(E–H)**, shows reduction in the number of lymphocytes and WBCs ranging from days 1-4 post-infection for each of the DENV serotypes tested. **(I–L)**, increase in the hematocrit (volume of packed red blood cells in the blood) for mice infected with DENV1-4. Hematocrit was significantly higher post-DENV infection for all the time points collected, however trending to be close to baseline levels by day 14. Significance was determined by comparison to baseline values (day 0) by 1-way ANOVA and is shown by * for p<0.05, ** for p<0.01, *** for p<0.001 and **** for p<0.0001, ns, not significant, compared to the baseline. Baseline values are shown as day 0.

Having shown that DENV serotypes 1-4 can replicate in various tissues in WT mice and induce transcriptional reprogramming consistent with infection, we sought to describe any pathology associated with infection. In humans, DENV disease is often associated with sudden drop in platelet counts (thrombocytopenia), leukopenia, and an increase in the hematocrit, or volume of packed red blood cells in the blood, which results from plasma loss from the circulation (29). To assess hematological changes during infection, mice were infected with DENV1-4 as before, followed by collection of blood at various time points post-infection for complete cell count analysis. Mice given DENV1-4 experienced a drop in platelet counts over the course of infection (**Figures 4A–D**). The platelet drop was sudden (24h) in the case of DENV1-3 infections (**Figures 4A–C**), however for DENV4, the drop in platelets was trending downward starting day 2 post-infection and significantly reduced by day 3 (**Figure 4D**). Similarly, reduced white blood cell (WBC) counts were observed for mice infected with DENV1-4 and lymphocytes were the predominant cell type affected (**Figures 4E–H**), supporting that these animals experience leukopenia during DENV disease. The numbers of monocytes and granulocytes did not consistently or significantly differ during the time course of DENV disease (**Figures 4E–H**). All the animals restored normal blood cell counts by day 14 post-infection (**Figures 4E–H**), consistent with the clearance of DENV by this time point in most animals (**Figure 1**). As reported previously only for DENV2 (11), hematocrit values were significantly elevated in mice infected with all four DENV serotypes (**Figures 4I–L**). Hematocrit was highest at days 1 and 2 post-infection, coinciding with the peak virus infection (**Figure 1**). Interestingly, hematocrit values remained high for a longer period of time and did not recover to baseline values until after infection cleared (**Figures 4I–L**). Overall, the data presented in **Figure 4** show that WT mice infected with DENV1-4 experience hematologic changes reminiscent of dengue disease.

### DENV-Induced Tissue Damage in the Liver

**Figure 5:**
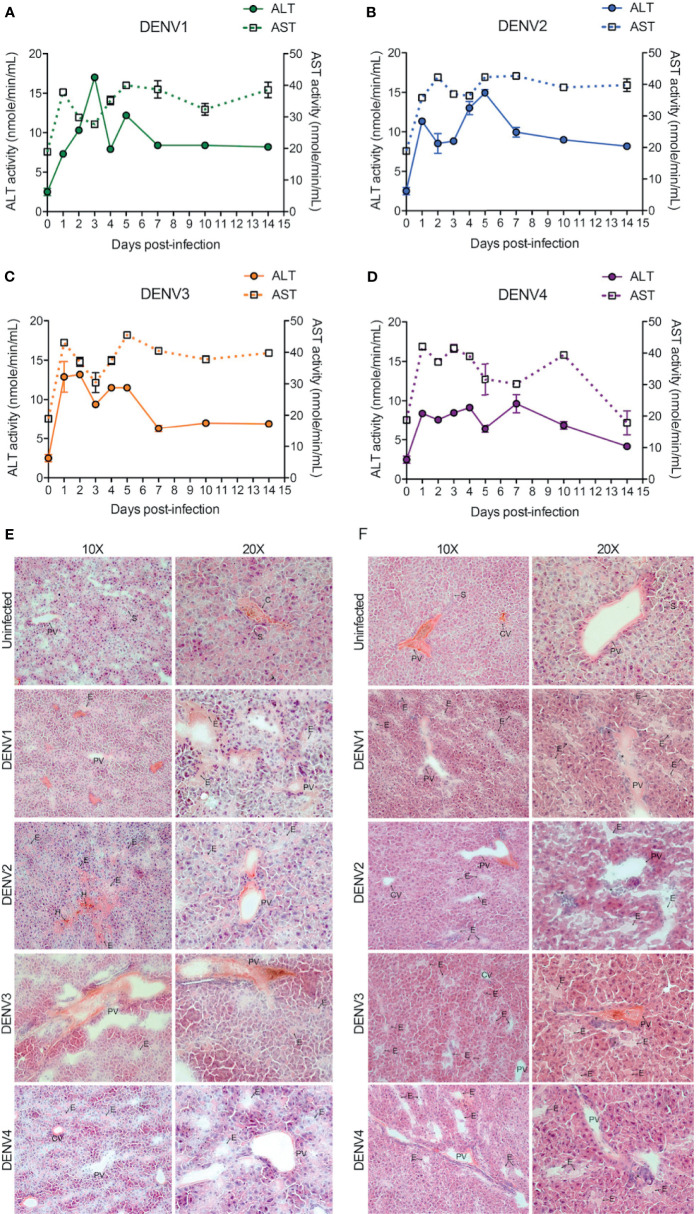
DENV infection causes liver damage in WT mice. **(A–D)**, Quantification of functional liver enzymes, alanine transaminase (ALT) and aspartate transaminase (AST) in the mice infected with DENV1-4. The activity levels of both ALT and AST enzymes were elevated up on DENV infection. Baseline values are shown as day 0. **(E, F)**, shows gross histological observations (hematoxylin and eosin stained sections) where edema **(E)** was observed in the liver tissues at days 2 **(E)** and 4 **(F)** post-infections. Abnormal hemorrhaging (H) and cellular infiltrates (*) were also observed for DENV2 infected liver tissues. Central vein (CV), portal vein (PV), and some sinuses (S) are labelled.

The liver is one of the major organs targeted by DENV and liver dysfunction and/or liver tissue damage have been consistently reported during DENV infection in humans (30, 31). To test whether liver functions were influenced during DENV infection in our mouse model, we measured the activity of aspartate transaminase (AST) and alanine transaminase (ALT) in the mouse serum. AST and ALT activity were significantly increased during DENV1-4 infections compared to the mock-infected healthy control group (**Figures 5A–D**). Interestingly, for DENV1-3, AST values remained elevated and did not normalize until day 14 post-infection, suggesting a strong tissue damage caused by DENV infection or DENV-induced inflammation in the liver (**Figures 5A–C**). For DENV-4, AST values returned to normal baseline levels by day 14 (**Figure 5D**), consistent with the evidence for limited liver infection in DENV4-infected animals (**Figure 1L**). Similarly, ALT levels were highest during the peak replication period (days 1-5) and then returned to baseline levels by day 14 (**Figures 5A–D**). AST and ALT levels also differed significantly between serotypes, with DENV1 reaching the highest peak ALT levels and DENV4 peaking the lowest (**Figures 5A–D**, **Supplementary Figure 7**). To verify that productive virus replication *in vivo* was required to induce elevated AST and ALT, we also measured these liver enzymes after injection of UV-inactivated virus, which was not capable of inducing liver enzymes (**Supplementary Figure 8**). These data suggest tissue damage or injury that is caused by acute DENV infection in WT mice. Further examination of liver injury was performed by tissue cross sectioning on days 2 and 4 post-infection, followed by histologic staining using hematoxylin and eosin. Edema with expansion of liver sinusoids was observed for DENV1-4 infections (**Figures 5E, F**). Hemorrhaging was also observed in case of DENV2-infected liver tissue (**Figure 5E**). At day 4 post-infection, infiltrations of mononuclear cells into the liver tissue were evident for all four serotypes of DENV infections (**Figure 5F**). Interestingly, we observed more prominent damage in DENV2-infected liver tissue compared to the DENV1, 3 and 4 infection groups. This observation is also consistent with peak infection titers concurrent with transcriptional enrichment of genes related to necrosis in the liver of DENV2-infected mice (**Figure 1J**, **Supplementary Figure 5**). Together our data demonstrate that liver pathologies are experienced by WT mice during the course of a DENV infection.

### Hemorrhaging and Infection of Multiple Cell Types in the DENV-Infected Spleen

**Figure 6:**
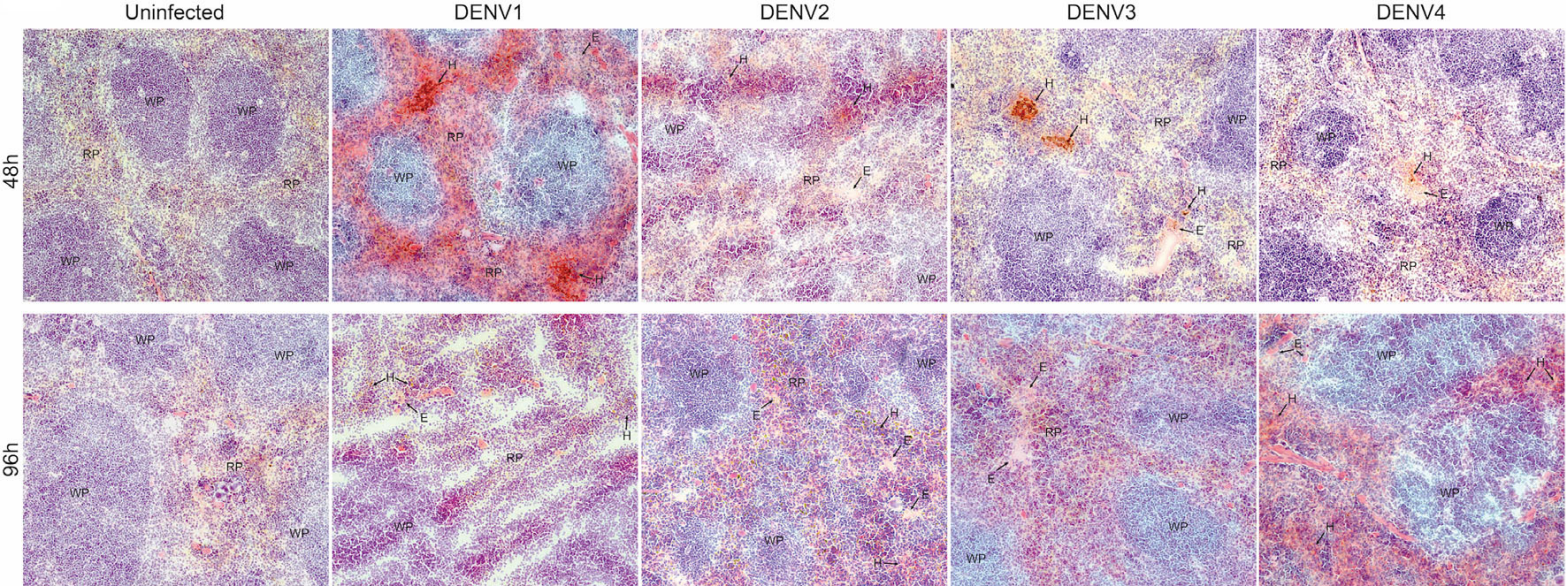
Hemorrhaging and edema in the spleen of mice infected with DENV1-4. Histological evidence of hemorrhaging (H) and edema (E) in the red pulp of the spleen of mice infected with DENV1-4 at 48h and 96h post-infection. Red pulp (RP) and white pulp (WP) are also labeled. Images were taken at 10x magnification and representative images from 3 animals are provided.

Due to the high burden of infection in the spleen (**Figure 1**), we also examined the infection and pathology of this organ in more detail. First, we observed splenomegaly, which peaked at differing time points for each serotype and coincided with an increase in the total cellularity of the spleen (**Supplementary Figure 9**). Interestingly, for some serotypes, there was a biphasic response with respect to splenic swelling (**Supplementary Figure 9A**), similar to the biphasic viral infection kinetics observed in the spleen (**Figure 1**). For all serotypes, hemorrhaging was also observed in the spleen (**Figure 6**). Splenic damage was much higher at 48h compared to 96h post-infection (**Figure 6**), which corresponds to the peak spleen virus infection, observed at days 1-2 for each DENV serotype, presented in **Figure 1**. These results support that pathological changes occur in the spleen that coincide with infection.

**Figure 7:**
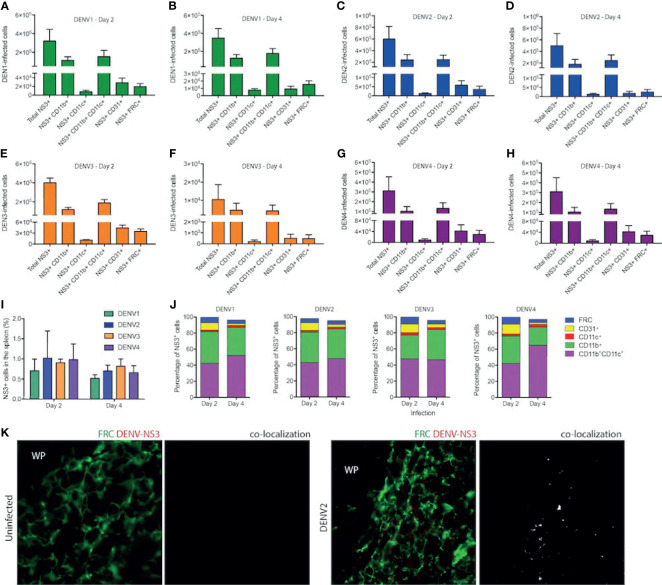
Cells of myeloid lineage and FRCs are the targets of DENV infection in WT mice. Numbers of cells infected in the spleens of mice after injection with DENV1 on **(A)** Day 2 **(B)** Day 4, DENV2 on **(C)** Day 2 and **(D)** Day 4, DENV3 on **(E)** Day 2 and **(F)** Day 4, and DENV4 on **(G)** Day 2 and **(H)** Day 4. **(I)** Percentages of total splenocytes that are NS3^+^ on Days 2 and 4 for each serotype. **(J)** Phenotypes of infected splenocytes as a percentage of NS3^+^ cells on days 2 and 4 post-infection. **(K)** Spleen sections stained for FRCs and DENV-NS3 protein for uninfected and DENV2-infected mice are presented. Co-localization of FRC and DENV-NS3 staining can be observed for DENV2-infected but not uninfected sections. White pulp location is indicated by (WP).

Since DENV1-4 could replicate and induce immune pathology in the WT mouse model in the spleen, we next aimed to identify the cell types infected *in vivo*. For this, we infected mice with DENV1-4 and isolated splenocytes for staining at either day 2 or 4 post-infection to detect the total numbers of various cell types during infection by each DENV serotype (**Figures 7A–H**). Splenocytes were stained to reveal the following cell subsets by surface markers: monocyte/macrophages (mo/MΦ, CD11b^+^CD11c^-^), monocyte-derived DCs (moDC, CD11b^+^CD11c^+^), conventional DCs (cDC, CD11b^-^CD11c^+^), endothelial cells (CD31^+^), and fibroblast reticular cells (FRC, antibody clone ER-TR7) (for gating strategy, see **Supplementary Figure 10**). Intracellular staining for DENV NS3 protein was used to detect infected cells. On day 2, for all serotypes of DENV1-4, approximately 1% of cells in the spleen were NS3^+^ (**Figure 7I**), corresponding to a total of approximately 3x10^6^ to 6x10^6^ infected cells (**Figures 7A–H**). On day 4, total numbers of infected cells ranged from 1x10^4^ for DENV3 (**Figure 7F**) to 5x10^5^ for DENV2 (**Figure 7D**) and moderate declines in the percentages of infected cells from day 2 were not significant (**Figure 7I**). A close examination of the subsets of cells infected in the spleen on day 2 revealed that most infected cells were CD11b^+^, either mo/MΦ or moDCs (**Figures 7A, C, E, G, J**). This is consistent with observations in humans that both mo/MΦ and DCs are highly permissive to infection. cDCs were also targets of infection accounting for 2-3% of infected cells (**Figure 7J**), perhaps due to the fact that cDCs are a minority cell population in the spleen (32); however, in spite of this they appear highly susceptible to infection (**Figures 7A–H, J**). The remaining cell types that were observed to be infected were FRCs and endothelial cells (**Figures 7A–H, J**), both constituting a small minority of the infected cells.

On day 4 of infection, the cell types infected remained largely the same for all serotypes (**Figures 7A–H, J**). Interestingly, when the percentages of each cell type infected were compared between days 2 and 4, we did observe some significant changes in the proportions of cells infected (**Figure 7J**). For example, for all serotypes, there was a reduction in the percentage of endothelial cells that were infected as a portion of the total cells (**Figure 7J**). For DENV3 there was an increase in the proportion of CD11b^+^ CD11c^-^ monocytes from day 2 to day 4 (**Figure 7J**). These are likely infiltrating cells, potentially infected, and may be responsible for prolonged infection of DENV3 in the spleen and liver (**Figure 1**). Notably, this data is also consistent with transcriptional signals associated with immune cell trafficking in the DENV3-infected spleen and liver (**Supplementary Figure 6**). For DENV1 and DENV4 infections, there was an increase in the percentage of infected cells that were moDCs (**Figure 7J**). To validate the novel observation of FRC infection *in vivo* by DENV, we stained spleen tissue sections for DENV protein NS3 in combination with an antibody against FRCs. While NS3 staining could not be observed in uninfected tissues, NS3 could be detected in the spleen in regions co-localizing with FRCs (**Figure 7K**), supporting that they are infected. These results demonstrate that the types of cells infected in the immune-competent mouse model recapitulate the human cell types that are thought to be infected during DENV infection and highlight that FRCs of the spleen are a previously unidentified target of DENV infection *in vivo*.

## Discussion

A growing body of literature has cautiously challenged the assumption that immune-competent mice are a poor model for DENV disease, for example, by showing that mild vascular leakage occurs during DENV infection or that low passage clinical isolates of DENV can be detected *in vivo* at early time points (9–13). Infection of C57Bl/6 mice with DENV2 has been shown to result in elevated liver enzymes both during primary and secondary infections (9). Another study showed that C57Bl/6 mice experienced thrombocytopenia, which is a hallmark of DENV infection, although they failed to detect infection by RT-PCR (10). We previously reported that C57Bl/6 mice experience replicating DENV infection in the liver and spleen after systemic inoculation, and in the draining lymph node after peripheral inoculation (11, 12). We also observed hemoconcentration and thrombocytopenia during systemic DENV2 infection (11). However, in spite of isolated reports, the use of immune-competent animals has continued to be highly controversial. Thus, we aimed here to systematically test the ability of DENV1-4 to induce sustained, replicating and systemic infection in immune-competent mice. In doing so, we found consistent, efficient virus replication *in vivo*, and signs of infection analogous to clinical dengue disease. These include hematological perturbations, hemorrhagic manifestations in target organs, transcriptional responses characteristic of a protective immune response or tissue damage, and multiple cell types infected *in vivo*.

For all of the clinical isolate DENV strains of serotypes 1-4 that were used here (Eden1-4), we observed systemic infection that could be detected in the spleen and liver for approximately 1-2 weeks. Replication kinetics showed two peaks in the spleen, peritoneum and liver, suggesting multiple rounds of replication occurred before clearance. We were able to validate that DENV is present in the serum and that virus can be detected by negative-strand PCR. Various controls were used throughout, including UV-inactivated virus and saline control groups and infection levels were compared to more permissive immunocompromised mice. Furthermore, high levels of secreted NS1 protein in the serum confirm active DENV replication in mice by a third measure. Infection of mice subcutaneously with DENV2 to model the natural route of infection was also effective in establishing systemic infection, although a high inoculation titer was required. Future studies will be needed to determine if this peripheral inoculation route is also feasible for strains from other serotypes. Interestingly, we observed that DENV2 and DENV3 induced the highest infection burden in tissues. In humans, DENV1-3 have also been shown in some studies to reach higher titers *in vivo* compared to DENV4 (33). However, it is important to note that virus genome copies for DENV are approximately 100-fold more abundant than infectious particles and that genome copy numbers to infectious particle ratios can also vary strain to strain (34, 35).

Here, we used relatively low passage (less than passage 10) clinical isolates and this resulted in consistent infection; however, in agreement with the literature, the NGC laboratory strain also did not lead to replicating infection *in vivo* in our hands. Thorough characterization of various DENV-target cell populations in the spleen of infected animals demonstrated strong replication in the cells of myeloid origin such as monocytes, macrophages and monocyte-derived DCs. Small proportions of conventional DCs and endothelial cells were also infected in the spleen. These cell populations are consistent with those that have been described as target infection cell types for humans (36) as well as shown using interferon receptor-deficient animals (37). In humans with DENV, endothelial cells have been previously reported to contain NS3 antigen (38, 39). We also now report a novel cellular target for DENV infection, FRCs of the spleen. Although FRCs have never been reported as targets of DENV *in vivo*, human fibroblasts are permissive to DENV infection in cell culture (40). Our results substantiate that FRCs are, indeed, true targets of infection *in vivo*. Interestingly, FRCs form the structural basis for the reticular channels that dendritic cells use to migrate within lymphoid tissues (41) and we expect that this could be a reservoir leading to infection of new DCs as they migrate within the spleen. Alternatively, FRCs could become infected themselves in the course of providing conduits for DC migration.

We found that our DENV4 isolate showed the least infection in the liver compared to the other serotypes with not all individual mice having detectable DENV4 in their livers. Consistent with this, AST values for DENV4-infected mice also returned to normal levels earlier than for the other serotypes. We also note that some independent studies have suggested DENV4 is the least likely serotype to cause liver pathology (42, 43), although, others have observed DENV4 infection associated with high levels of serum liver enzymes (44). Serotype-specific attachment of DENV to liver cells has also been described (45). However, the effects of DENV on the liver could also be in-part immune mediated and indirect. We previously reported that transcriptional activation of immune pathways in the liver could be suppressed using an immune modulatory drug that limits mast cell activation (13). This supports the assumption that pathology in tissues may be due to the actions of immune cells in addition to direct effects of virus replication and could potentially explain why liver enzyme levels don’t always correlate with virus titer in that organ. However, virus replication or amplification *in vivo*, either in the liver or another target organ, is required for elevated liver enzymes since UV-inactivated virus did not induce them. In contrast to DENV4, DENV2 appeared to induce the most tissue damage in the liver. Not only did we observe “liver necrosis” as a key pathway enriched in DENV2-infected liver according to our transcriptional analysis, we also observed DENV2-induced hemorrhaging in the liver tissue.

The transcriptional profiling in the tissues illustrates that infection induces a wide array of host-response genes that are characteristic of viral infection control, such as the IFN response pathway, which was one of the most significantly perturbed pathways. We noted that the transcriptional response closely tracked with the viral infection kinetics. For example, during DENV1 infection where the infection peaked later, so did the transcriptional response. Interestingly, there were also differences in viral kinetics that could not be explained entirely by IFN response pathways. For example, infection by our DENV3 strain persisted longer in both the spleen and liver than the 3 strains representative of other serotypes, in spite of efficient induction of IFN response genes. This could suggest that this DENV3 strain has alternative mechanisms of suppressing the host antiviral response outside of targeting IFN pathway, a question that warrants future study.

Histological observations in the liver and spleen of all serotypes of DENV-infected mice also revealed dengue pathologies consistent to those observed in humans (39), including micro-hemorrhaging. The observed damage to the liver was further solidified by detection of elevated liver enzymes in the serum, ALT and AST (46). Humans with dengue disease display an altered hematological profile involving leukopenia and thrombocytopenia in most cases, whether mild or severe (47, 48). Complete blood cell count analysis of mice infected with DENV1-4 in this study demonstrated pathologies consistent with these. For example, we observed significantly elevated hematocrit levels and drops in platelets coinciding with the window of active viral replication. We observed a significant reduction of WBC and lymphocytes, which also occurs during human dengue disease (49). In particular, elevated hematocrit is an important non-invasive measure that indicates hemoconcentration during infection and it is often observed in DENV patients that are experiencing plasma loss (50). Our results also indicate that mice experience significant and prolonged hemoconcentration for several days post-infection with each of the clinical isolate strains used in this study. DENV disease kinetics in mice were consistent with an acute disease. Since these animals have fully functional immune system, we also observe that they clear DENV after the course of acute infection, as do humans.

In spite of the long-held assumption that DENV does not replicate in WT mice there has been little primary evidence supporting this claim. Hence, our understanding of the extent of viral replication and symptoms of dengue disease in immune-competent hosts has been lacking. Our data shows that the WT mouse model is, indeed, permissive to DENV infection with replication kinetics, transcriptional activation, cellular targets of infection and clinical signs consistent with DENV disease in humans. While certain studies requiring more robust replication or a lethal model, such as for antiviral drug testing, may be better suited for the traditional immune-compromised models, the use of this validated WT mouse model can contribute substantially to our understanding of dengue immunity and pathogenesis.

## Data Availability Statement

The gene expression dataset is available through NCBI GEO database (GEO submission number: GSE174807). The original contributions presented in the study are included in the article and associated **Supplementary Material**. Further inquiries can also be directed to the corresponding authors.

## Ethics Statement

The animal study was reviewed and approved by SingHealth IACUC.

## Author Contributions

The project was conceived by AR and AS. Experiments were performed by CM, MT, AR and SA. Transcriptomics and pathway analysis were performed by JM and RS. The manuscript was written by AR, JM and AS. All authors contributed to the article and approved the submitted version.

## Funding

This work was funded by NMRC/CBRG/0084/2015 and a New Investigator Grant 1054/2011 from the National Medical Research Council of Singapore and Start-up funding from Duke-NUS Medical School.

## Conflict of Interest

The authors declare that the research was conducted in the absence of any commercial or financial relationships that could be construed as a potential conflict of interest.
