# Supplementary material for: Immunological and Pathological Landscape of Dengue Serotypes 1-4 Infections in Immune-Competent Mice
Source: Front Immunol. 2021 Jun 8;12:681950. doi: 10.3389/fimmu.2021.681950 (PMC8219075; doi:10.3389/fimmu.2021.681950)
Supplement: Supplementary file 1 [file DataSheet_1.pdf]

Supplementary Table 1: List of primers and probes used in this study for quantification of the DENV genome in the mouse tissues.

	cDNA synthesis primer	qPCR primers
**DENV1**		
Peritoneal cells	5’-CTGAGTGAATTCTCTCTACTGAACC-3′	5’-CAAAAGGAAGTCGTGCAATA<br>5’-CTGAGTGAATTCTCTCTACTGAACC
Spleen	5’-CTGAGTGAATTCTCTCTACTGAACC-3′	5’-CAAAAGGAAGTCGTGCAATA<br>5’-CTGAGTGAATTCTCTCTACTGAACC
Liver	5’-CTGAGTGAATTCTCTCTACTGAACC-3′	5’-CAAAAGGAAGTCGTGCAATA<br>5’-CTGAGTGAATTCTCTCTACTGAACC
**DENV2**		
Peritoneal cells	5’-TTGCACCAACAGTCAATGTCTTCAGGTTC-3′	5’-TCAATATGCTGAAACGCGCGAGAAACCG-3′<br>5’-CGCCACAAGGGCCATGAACAG-3′
Spleen	5′-CCCATCTCITCAIIATCCCTGCTGTTGG-3′	5’-AATATGCTGAAACGCGAGAGAAACCGCG-3’<br>5’-CCCATCTCITCAIIATCCCTGCTGTTGG-3′<br>Probe:<br>5’-AGCATTCCAAGTGAGAATCTCTTTGTCAGCTGT-3’
Liver	5′-CCCATCTCITCAIIATCCCTGCTGTTGG-3′	5’-AATATGCTGAAACGCGAGAGAAACCGCG-3’<br>5’-CCCATCTCITCAIIATCCCTGCTGTTGG-3’<br>Probe:<br>5’-AGCATTCCAAGTGAGAATCTCTTTGTCAGCTGT-3’
Serum	5’-TTGCACCAACAGTCAATGTCTTCAGGTTC-3’	5’-TCAATATGCTGAAACGCGCGAGAAACCG-3′<br>5’-CGCCACAAGGGCCATGAACAG-3′
**DENV3**		
Peritoneal cells	5′-CCCATCTCITCAIIATCCCTGCTGTTGG-3′	5’-AATATGCTGAAACGCGAGAGAAACCGCG-3′<br>5′-CCCATCTCITCAIIATCCCTGCTGTTGG-3′<br>Probe:<br>5’-AGCATTCCAAGTGAGAATCTCTTTGTCAGCTGT-3’
Spleen	5’-CTCTAGATGTCAGCTTTCTTAG-3′	5’- GCGCACACACTAATAATGA-3′<br>5’- CTCTAGATGTCAGCTTTCTTAG-3′
Liver	5’-TTGCACCAACAGTCAATGTCTTCAGGTTC-3′	5’- TCAATATGCTGAAACGCGCGAGAAACCG-3′<br>5’- TAACATCATCATGAGACAGAGC-3′
**DENV4**		
Peritoneal cells	5’- TCCACCTGAGACTCCTTCCA-3′	5’- TTGTCCTAATGATGCTGGTCG-3′<br>5’- TCCACCTGAGACTCCTTCCA-3′
Spleen	5’- TCCACCTGAGACTCCTTCCA-3′	5’- TTGTCCTAATGATGCTGGTCG-3′<br>5’- TCCACCTGAGACTCCTTCCA-3′
Liver	5’- GTTGGGTTCACTTGAGAATAAC-3′	5’-GCTGAGACACACCATAGAA-3′<br>5’- GTTGGGTTCACTTGAGAATAAC-3′
**DENV2 -ve strand**	5’-AATATGCTGAAACGCGAGAGAAACCGCG-3’	5’-AATATGCTGAAACGCGAGAGAAACCGCG-3’<br>5’-CCCATCTCITCAIIATCCCTGCTGTTGG-3′

Figure S1: **Validated specific DENV detection in infected tissues by PCR.** Gels of PCR products are presented to demonstrate that real time PCR detection of virus *in vivo* was specific for (A) DENV1 (expected size: 111 bp) (B) DENV2 (expected size: 118 bp) (C) DENV3 (expected size: 170 bp) and (D) DENV4 (expected size: 88 bp). For all gels, Lane 1: Marker, Lane 2: positive control plasmid containing the region of interest, Lane 3: Negative control tissue, Lane 4: Infected tissue.

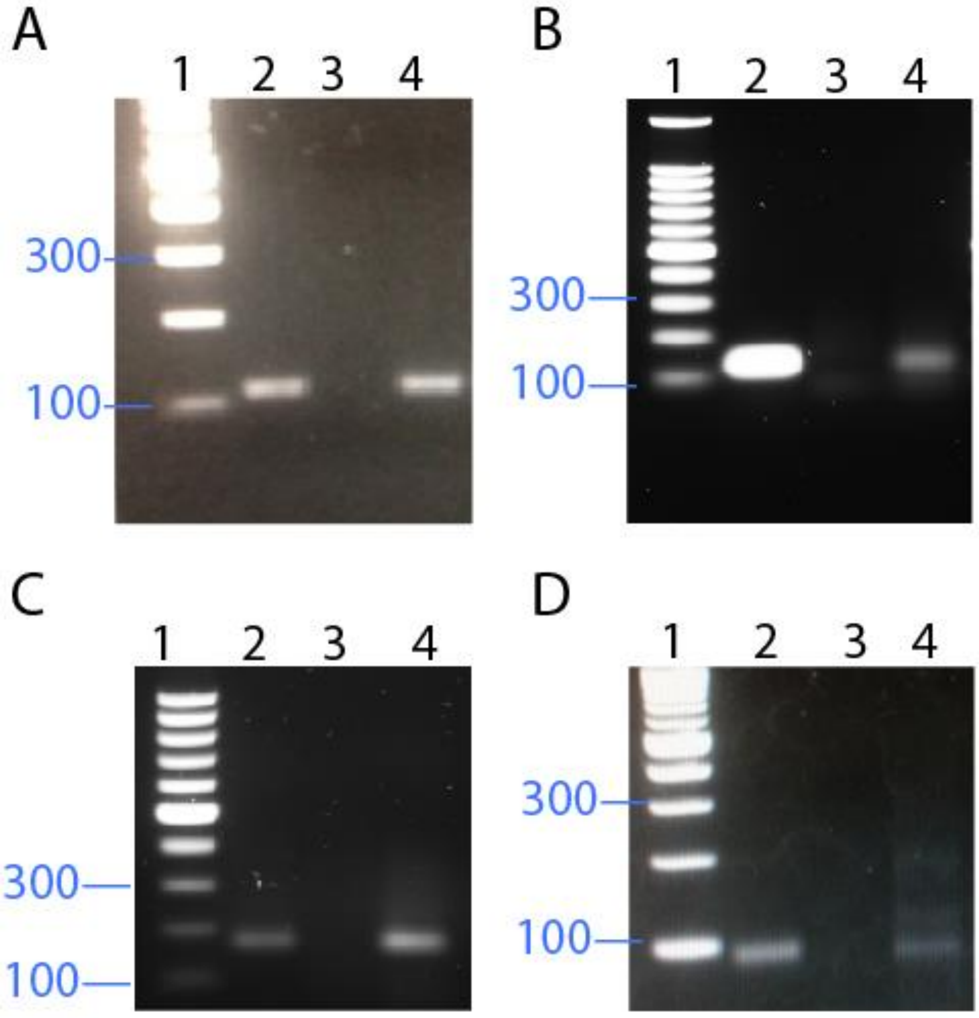

Figure S2. Comparison of viral burden between wild-type and immunocompromised mice. DENV2 infection is lower in WT (C57Bl/6) mice in comparison to interferon-deficient AG129 mice. C57Bl/6 or AG129 mice were injected i.p. with 1x10^6 pfu of DENV2 or UV-inactivated DENV2. After 24 or 48h, DENV2 genome copies were quantified by RT-PCR in (A) peritoneal cells, (B) spleen, (C) serum and (D) liver. Although comparatively lower DENV infection in WT mice than AG129 mice, the detection of virus replication is specific. For instance when C57Bl/6 WT mice were injected with UV-inactivated virus, input virus was only detected at the site of inoculation in the (A) peritoneum and not at any other secondary sites (B-D).

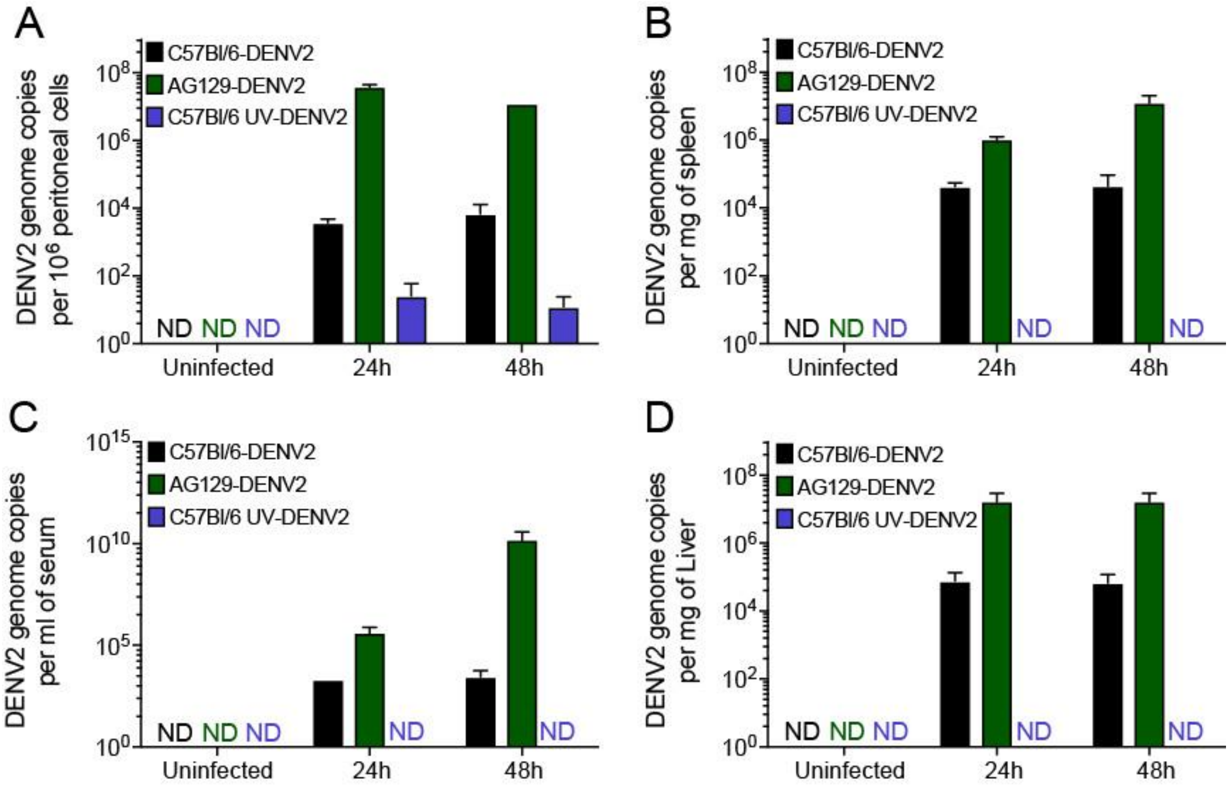

Figure S3. Detection of DENV in subcutaneously infected WT mice. Mice were infected subcutaneously in rear foot pads (FP) with 8x10^{8} pfu of DENV2 virus. DENV2 genome copy numbers were quantified in the (A) FP skin, spleen, liver, (B) draining lymph node (DLN), and (C) Serum at 24, 48 and 72h post infection by RT-PCR.

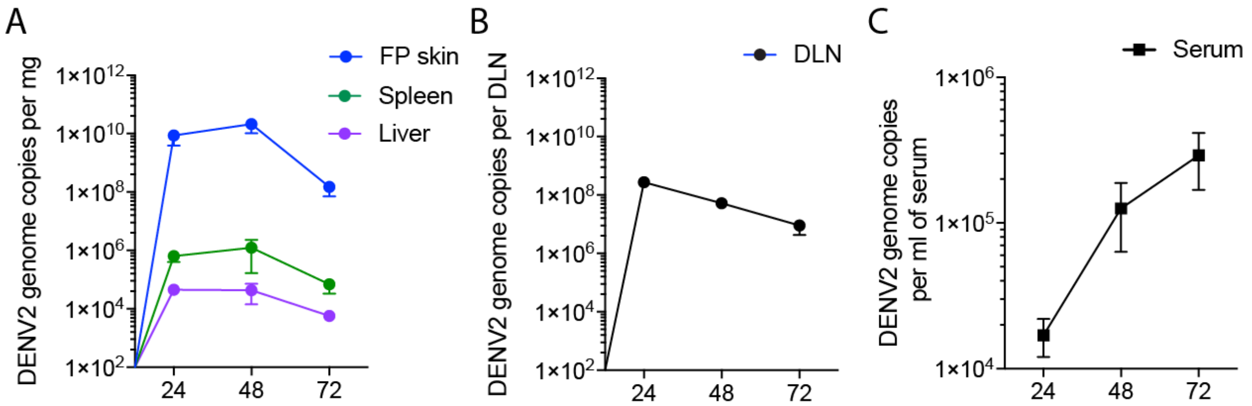

Figure S4: **Detection of DENV2 negative-strand by PCR.** Gel image showing detection of DENV2 negative-strand RNA in tissue samples including PBMCs, cells from the peritoneal lavage (PL), spleen, and liver 24h post-infection with DENV2, or injection of an equal amount of UV-inactivated DENV2 (DENV2-UV). Negative-strand PCR was performed according to published methods[1-3].

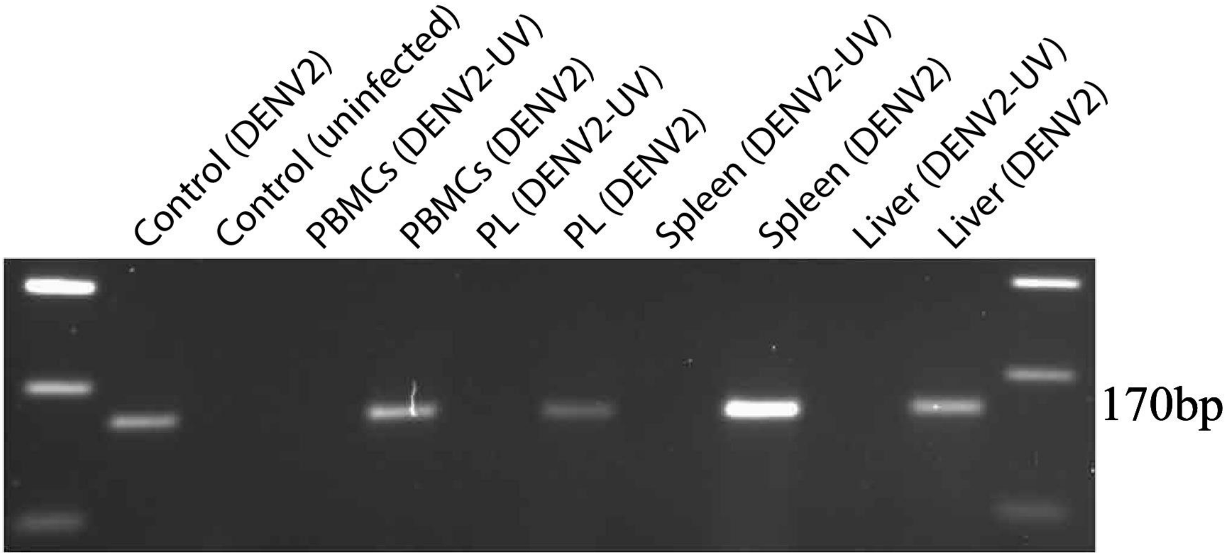

Figure S5: **“Necrosis of liver” pathway is enriched for DENV2.**

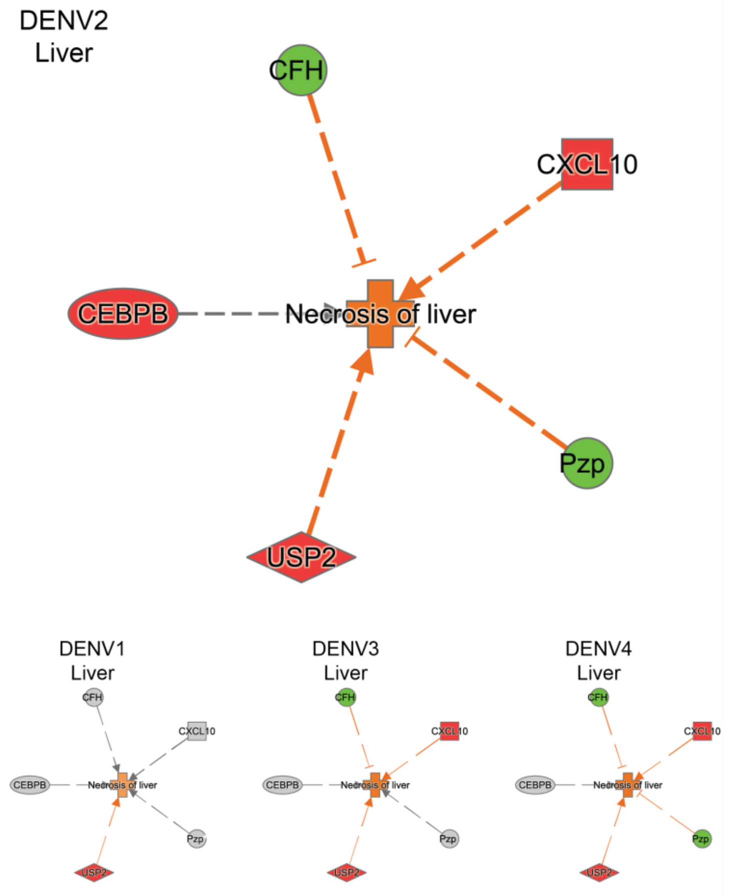

Figure S6: Cellular trafficking changes associated with DENV3 infection. Pathway analysis revealed significant perturbation of cellular trafficking associated pathways with (A) DENV3 infection in the liver, “mobilization of blood cells” and (B) in the spleen, “migration of cells”.

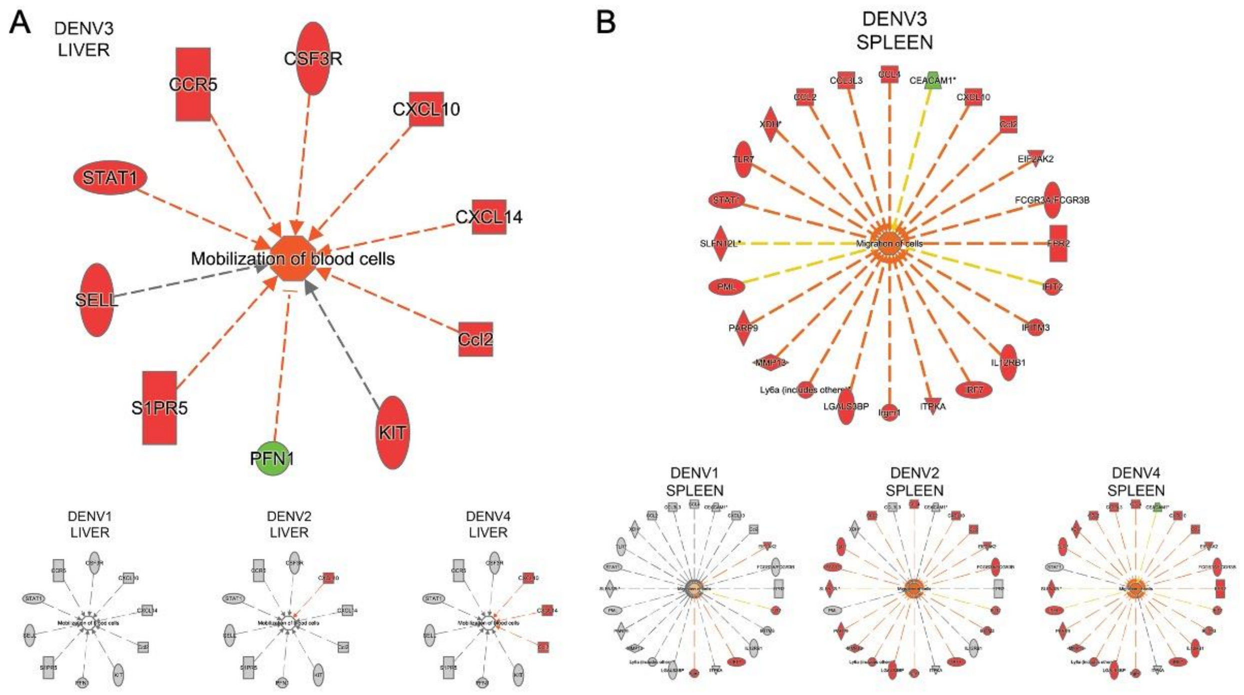

Figure S7: Comparison of AST and ALT among DENV1-4 groups. (A) ALT and (B) AST levels differed significantly among groups of mice infected with DENV1-4, as determined by 2-way ANOVA ($p<0.0001$) for both panels. This figure is an alternate presentation of data contained in Figure 5A-D.

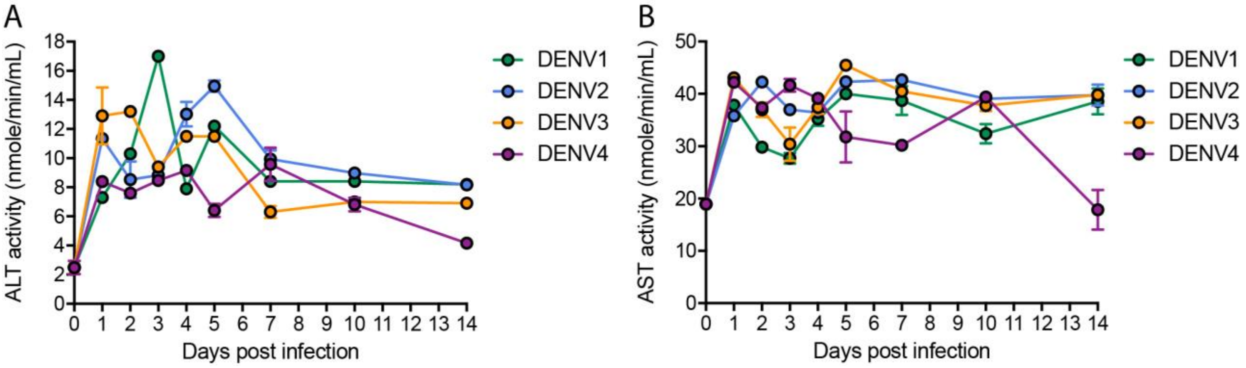

Figure S8: UV-inactivated DENV does not induce liver enzymes. A) ALT and (B) AST levels did not differ significantly from baseline in animals injected with UV-inactivated DENV2.

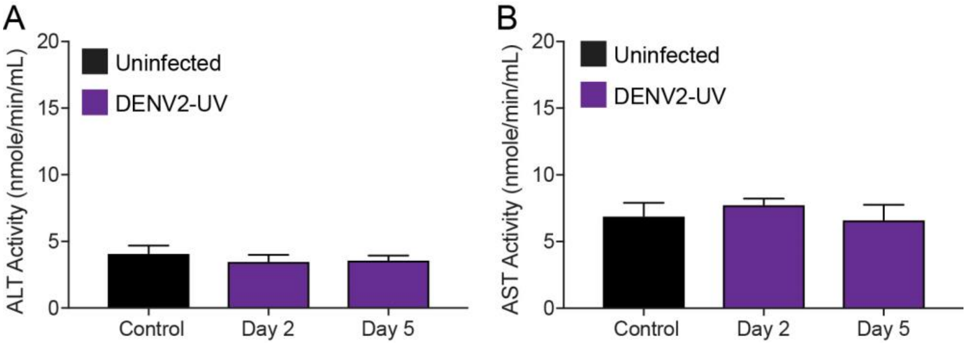

Figure S9: **Splenomegaly in immune-competent WT mice after infection with DENV serotypes 1-4.** Graphs depict the (A) spleen mass and (B) Total splenocytes at indicated times points post-infection. For (B), significance was determined by comparison to uninfected control using students unpaired t-test and is shown by * for p<0.05, ** for p<0.01 and *** for p<0.001.

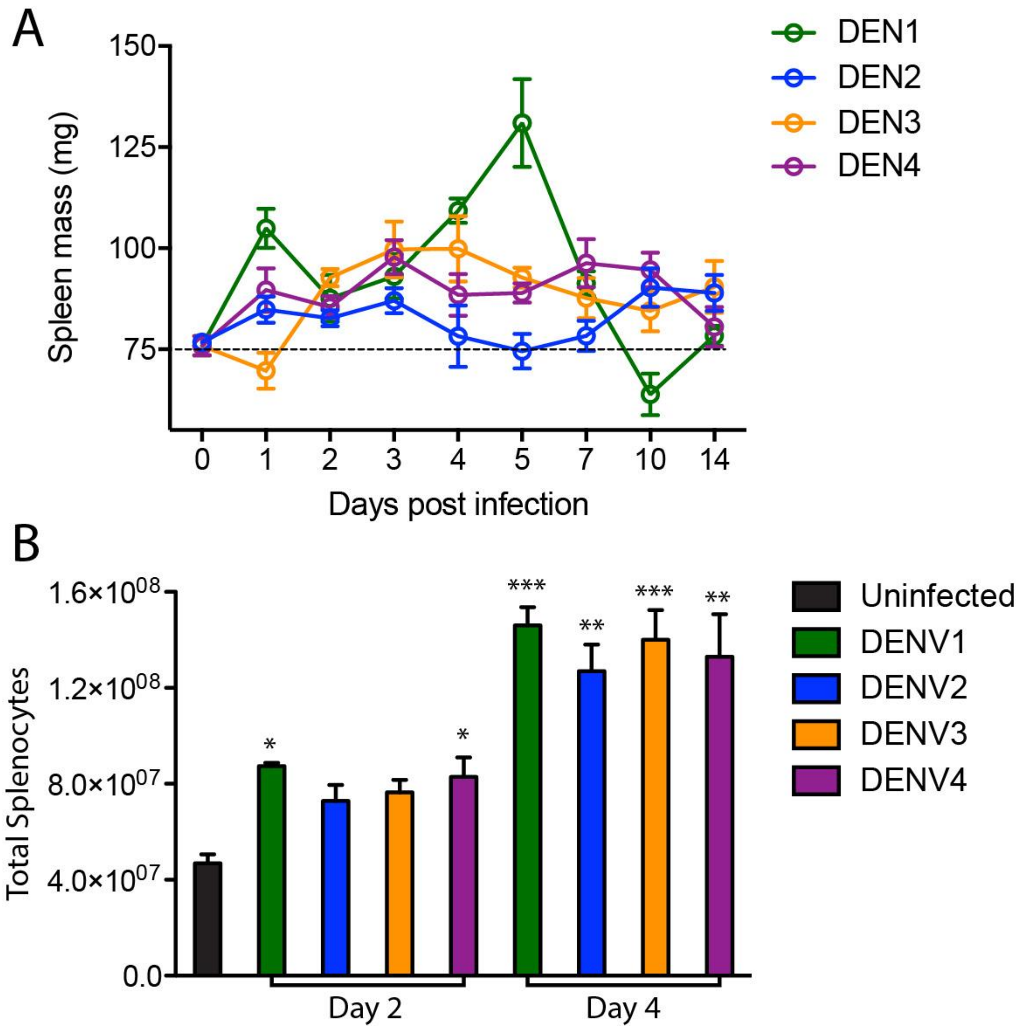

Figure S10: Flow cytometry gating strategy to identify DENV-infected cell populations in the spleen. Intracellular staining with DENV NS3[+] was used in conjunction with staining for phenotypic markers CD11b, CD11c, CD31, and FRC antigen to define infected cell types. Isotype and uninfected controls are also presented to show specificity of staining.

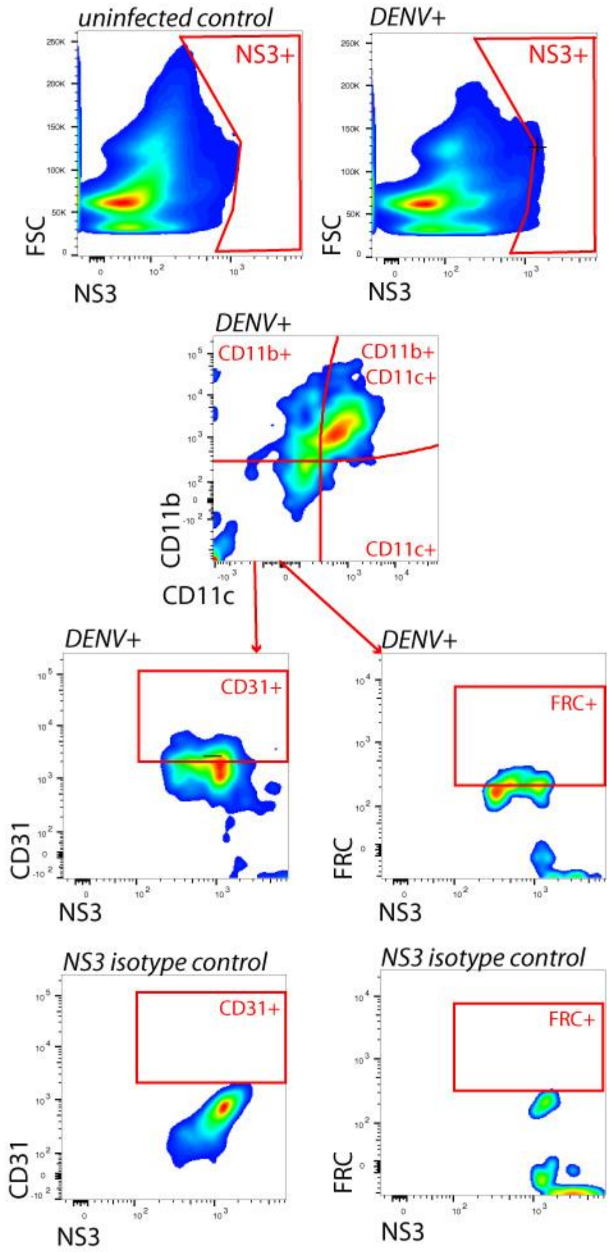

1 Wang, W. K., Lee, C. N., Kao, C. L., Lin, Y. L. & King, C. C. Quantitative competitive reverse transcription-PCR for quantification of dengue virus RNA. *J Clin Microbiol* **38**, 3306-3310 (2000).

2 Wang, W. K. *et al.* Detection of dengue virus replication in peripheral blood mononuclear cells from dengue virus type 2-infected patients by a reverse transcription-real-time PCR assay. *J Clin Microbiol* **40**, 4472-4478 (2002).

3 Morrison, J. *et al.* Transcriptional Profiling Confirms the Therapeutic Effects of Mast Cell Stabilization in a Dengue Disease Model. *J Virol* **91**, doi:10.1128/JVI.00617-17 (2017).
